# Supplementary material for: A Global Screen for Assembly State Changes of the Mitotic Proteome by SEC-SWATH-MS
Source: Cell Syst. 2020 Feb 26;10(2):133–155.e6. doi: 10.1016/j.cels.2020.01.001 (PMC7042714; doi:10.1016/j.cels.2020.01.001)

P48553 | TPC10_HUMAN | TRAPPC10 EHOC1 TMEM1
Monomer MW [kDa]: 142.189 Monomer expected elution fraction: 37

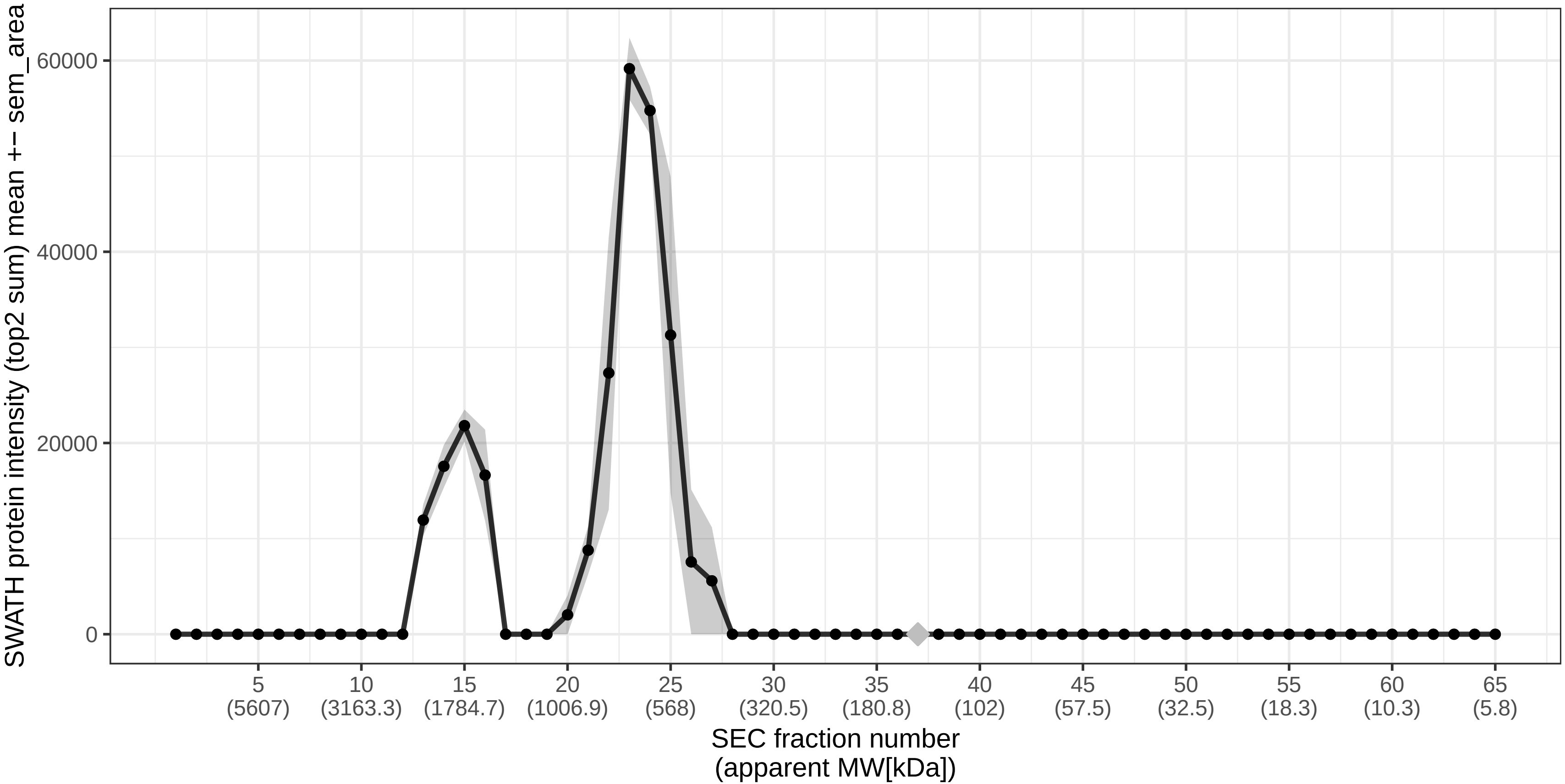

Supplement: Data S1. SEC-SWATH-MS Protein Chromatograms, Related to Figure 1 [file mmc6.zip › SECchrom_P48553_TPC10_HUMAN_TRAPPC10_EHOC1_TMEM1.pdf]
